# A machine learning-based model to predict POD24 in follicular lymphoma: a study by the Chinese workshop on follicular lymphoma

**DOI:** 10.1186/s40364-024-00716-4

**Published:** 2025-01-03

**Authors:** Jie Zha, Qinwei Chen, Wei Zhang, Hongmei Jing, Jingjing Ye, Huanhuan Liu, Haifeng Yu, Shuhua Yi, Caixia Li, Zhong Zheng, Wei Xu, Zhifeng Li, Zhijuan Lin, Lingyan Ping, Xiaohua He, Liling Zhang, Ying Xie, Feili Chen, Xiuhua Sun, Liping Su, Huilai Zhang, Haiyan Yang, Weili Zhao, Lugui Qiu, Zhiming Li, Yuqin Song, Bing Xu

**Affiliations:** 1 Department of Hematology, The First Affiliated Hospital of Xiamen University and Institute of Hematology, School of Medicine, Xiamen University, Xiamen, 361003 P.R. China; 2 Key laboratory of Xiamen for diagnosis and treatment of hematological malignancy, Xiamen, China; 3 Department of Hematology, Peking Union Medical College Hospital, Beijing, China; 4 Department of Hematology, Lymphoma Research Center, Peking University Third Hospital, Beijing, China; 5 Department of Hematology, Cheeloo College of Medicine, Qilu Hospital, Shandong University, Jinan, China; 6 Department of Lymphoma, Cancer Hospital of the University of Chinese Academy of Sciences, Zhejiang Cancer Hospital), Hangzhou, China; 7 Department of Lymphoma, Institute of Cancer and Basic Medicine (IBMC), Chinese Academy of Sciences, Hangzhou, China; 8 State Key Laboratory of Experimental Hematology, National Clinical Research Center for Blood Diseases, Blood Diseases Hospital, Institute of Hematology, Chinese Academy of Medical Sciences and Peking Union Medical College, Tianjin, China; 9 National Clinical Research Center for Hematologic Diseases, Jiangsu Institute of Hematology, The First Affiliated Hospital of Soochow University, Suzhou, China; 10 Shanghai Institute of Hematology, State Key Laboratory of Medical Genomics, National Research Center for Translational Medicine at Shanghai, Ruijin Hospital Affiliated to Shanghai Jiao Tong University School of Medicine, Shanghai, China; 11 Department of Hematology, Jiangsu Province Hospital, The First Affiliated Hospital of Nanjing Medical University, Nanjing, China; 12 Key Laboratory of Carcinogenesis and Translational Research (Ministry of Education), Peking University Cancer Hospital and Institute, Beijing, China; 13 Department of Medical Oncology, Sun Yat-sen University Cancer Center, Guangzhou, China; 14 State Key Laboratory of Oncology in South China, Guangzhou, China; 15 Collaborative Innovation Center for Cancer Medicine, Guangzhou, China; 16 Cancer Center, Union Hospital, Tongji Medical College, Huazhong University of Science and Technology, Wuhan, China; 17 Department of Hematology, Fujian Provincial Hospital, Shengli Clinical Medical College of Fujian Medical University, Fujian Medical University, Fuzhou, China; 18 Lymphoma division, Guangdong Provincial People’s Hospital, Guangdong Academy of Medical Sciences, Guangzhou, China; 19 Department of Oncology, The Second Hospital of Dalian Medical University, Dalian, Liaoning China; 20 Shanxi Hospital Affiliated to Cancer Hospital, Cancer Hospital, Shanxi Province Cancer Hospital, Chinese Academy of Medical Sciences, Shanxi Medical University, Taiyuan, Shanxi China; 21 Department of Lymphoma, Tianjin Medical University Cancer Hospital, Tianjin, China

**Keywords:** Follicular lymphoma, POD24, Machine learning, FLIPI-C, Overall survival

## Abstract

**Background:**

Disease progression within 24 months (POD24) significantly impacts overall survival (OS) in patients with follicular lymphoma (FL). This study aimed to develop a robust predictive model, FLIPI-C, using a machine learning approach to identify FL patients at high risk of POD24.

**Methods:**

A cohort of 1,938 FL patients (FL1-3a) from seventeen centers nationwide in China was randomly divided into training and internal validation sets (2:1 ratio). XGBoost was utilized to construct the POD24-predicting model, which was internally validated in the validation set and externally validated in the GALLIUM cohort. Key predictors of POD24 included lymphocyte-to-monocyte ratio (LMR), lactate dehydrogenase (LDH) > ULN, low hemoglobin (Hb), elevated beta-2 microglobulin (β2-MG), maximum standardized uptake value (SUVmax), and lymph node involvement. The FLIPI-C model assigned 2 points to LMR and 1 point to each of the other variables.

**Results:**

The FLIPI-C model demonstrated superior accuracy (AUC) for predicting POD24 and 3-year overall survival (OS) in both the internal (AUC POD24: 0.764, OS: 0.700) and external validation cohorts (AUC POD24: 0.703, OS: 0.653), compared to existing models (FLIPI, FLIPI-2, PRIMA-PI, FLEX). Decision curve analysis confirmed the superior net benefits of FLIPI-C.

**Conclusions:**

Developed using a machine learning approach, the FLIPI-C model offers superior predictive accuracy and utilizes simple, widely available markers. It holds promise for informing treatment decisions and prognostic assessments in clinical practice for FL patients at high risk of POD24.

**Supplementary Information:**

The online version contains supplementary material available at 10.1186/s40364-024-00716-4.

## Introduction

Follicular lymphoma (FL) is characterized by an indolent course, with a median overall survival (OS) of more than 10–12 years. The therapeutic response and outcome of patients with FL are highly heterogeneous, with some experiencing poor outcomes due to eraly relapse or progression [1–3]. POD24, defined as disease recurrence or progression within 24 months of initial chemoimmunotherapy, occurs in approximately 20% of patients with FL, with the five-year OS rate ranging from 29 to 54% [4–7]. Thus, the precise identification of patients at risk for POD24 is the prerequisite for treatment decision-making to improve their outcome, as these patients may benefit from more intensive and effective therapies [8, 9]. However, it remains a major challenge to distinguish the patients with FL at high risk for POD24, particularly those with low tumor burden such as FL1-3a.

The capability of existing prognostic models to predicting the risk of POD24 in patients with FL has been investigated, showing that each of them has its inevitable limitations [10]. The follicular lymphoma international prognostic index (FLIPI) is currently used widely to identify high-risk patients with FL in daily practice, of whom only 56–58% experience POD24 [11, 12]. The PRIMA-prognostic index (PRIMA-PI) is easy to use, but its accuracy in predicting EFS24 is insufficient [13]. With inclusion of genetic features, the POD24-PI and m7-FLIPI perform better than the FLIPI in predicting POD24 [12, 14]. However, they require the information of gene mutations, which may not always be available in clinical practice. Of note, the accuracy of most existing models in predicting POD24 has not yet been evaluated in different ethic populations, including the Chinese population with FL.

Virtually all existing risk-scoring models for FL were developed using statistical approaches such as logistic regression or Cox regression [8, 12, 14, 15]. Recently, the rapid advance in machine learning facilitates the development of prognostic models to aid clinicians’ decision-making in various diseases, including FL [16–19]. For nonlinear date, the decision tree-based models, such as extreme gradient boosting (XGBoost) and deep neural networks, have been shown superiority to traditional linear models (e.g., logistic or Cox regression) in predictive capability [20, 21].

Taken advantage of a large cohort of FL patients, we aim to develop a reliable model for predicting POD24 in FL patients using a machine learning approach (XGBoost). The performance of this model, FLIPI-C, was then validated in an internal validation cohort as well as externally in the GALLIUM cohort. Its stability and reliability were compared with the existing prognostic models, including the FLIPI, FLIPI-2, PRIMA-PI, and FLEX.

## Patients and methods

### Patients

This retrospective, multicenter study included a cohort of patients newly diagnosed FL1-3a between January 2000 and December 2020 at seventeen centers nationwide in China. Patients were eligible if they were aged > 18 years and had a histological diagnosis of grade 1-3a FL according to the WHO classification. Among a total of 2,243 patients, patients with insufficient clinical information (*n* = 292) and loss of follow up (*n* = 13) were excluded. Eligible patients (*n* = 1,938) were randomly divided into a training set (*n* = 1,292) for model development and a validation set (*n* = 646) for internal validation. This study was approved by the institutional review boards of all participating institutions, and written informed consent was obtained from the participants. For external validation, the data of 1,145 patients who were eligible for this study were obtained from the GALLIUM study via the Vivli platform (http://vivli.org).

The data for this study included demographic and clinical characteristics at the time of initial diagnosis, including age, sex, performance status (PS), disease stage, histological grade, B symptoms, lymph node (LN) involvement, extranodal disease, bone marrow (BM) involvement, splenic involvement, presence of bulky disease, serum lactate dehydrogenase (LDH), hemoglobin (Hb), lymphocyte-to-monocyte ratio (LMR), maximum standardized uptake value (SUVmax), and $\:{\upbeta\:}$2-microglobulin (β2-MG).

### Outcomes

This study had a minimum follow-up of 2 years. POD24 was defined as disease progression or death due to lymphoma within the first 24 months after treatment initiation. PFS was defined as the time from diagnosis to progression, relapse, death due to any cause, or last follow-up. OS was defined as the time from diagnosis to death or last follow-up.

### Development of the POD24-predicting model (FLIPI-C)

We firstly build four binary classifiers (DecisionTree, RandomForest, RidgeClassifier and XGBoost). All classifiers included the same 15 selected features. Binary models used the boolean of POD24 status. For the predictor data we imputed missing values by randomForest (version 4.7) due to the varying degrees of tolerance for missing values among the four models. To minimize overfitting, the models underwent a stepwise-forward selection process to identify additional significant predictors. We compared the superiority and inferiority of the four models through the confusion matrix index (Supplementary Fig. 1). In the training cohort, we employed the permutation-based XGBoost algorithm to identify important variables associated with POD24. This algorithm ranked the features based on the variable importance metric of XGBoost and iteratively eliminated individual features to obtain the best prognostic model using SHAP analysis. Simultaneously, logistic regression was conducted to identify independent risk factors associated with POD24. Additionally, Cox regression analysis was performed to identify significant predictors associated with both PFS and OS. The predictors that showed importance in the SHAP analysis, logistic regression, and Cox regression were selected as the final set of variables. To enhance the explanatory power and facilitate clinical application, the coefficients derived from the XGBoost model to were utilized to create a scoring function. This scoring function aimed to assign scores to each variable based on their contribution to the prediction of POD24. This approach improves the interpretability and practicality of the model for use in clinical settings.

### Validation of the FLIPI-C

The FLIPI-C was then validated in the internal validation set and the external validation cohort, respectively. The performance of the FLIPI-C was also compared with the prognostic models currently used in clinical practice, including the FLIPI, FLIPI-2, PRIMA-PI, and FLEX, a scoring model reported recently [8].

To evaluate the discriminating ability of the FLIPI-C and the existing models, a time-dependent analysis of the area under the receiver operating characteristic curve (AUROC) were conducted. Briefly, we classified patients with binary observations (POD24 and 3-years OS) as either “positive” or “negative”. For each integer score in a model, the corresponding sensitivity (the probability that the model predicted a positive outcome when the observations were indeed positive) and specificity (the probability that the model predicted a negative outcome when the observations were indeed negative) were calculated. The ROC curve for each model was then created by plotting the pairs of sensitivity vs. specificity for every possible decision threshold. The area under the ROC curve (AUC) with 95% CIs was calculated to evaluate the ability of discriminating POD24 and 3-years OS. The clinical utility was evaluated using decision curve analyses (DCA). Considering the potential benefits and harms associated with different decision threshold, DCA could assist in determining the overall clinical usefulness of each model.

To assess the impact of risk stratification on PFS and OS, patients were categorized into high and low risk according to the FLIPI-C and the existing model. The optimal cut-off threshold in the ROC curve of POD24 was determined by Youden Index (the maximal sensitivity + the maximal specificity − 1). Kaplan-Meier curves were constructed by plotting the survival against time to compare the outcomes between low and high-risk groups.

### Statistical analysis

Demographic and clinical characteristics were compared between two groups using Pearson’s chi-square test (for categorical variables) or independent t-test (for continuous variables). Descriptive variables are presented as mean ± SD (for normally distributed data) or median with the corresponding 25th and 75th percentile (for skewed data). All statistical analyses were performed using R software version 4.0.3 (http://www.r-project.org). *P* < 0.05 was considered statistically significant.

## Results

### Baseline characteristics

The baseline clinical characteristics of the patients eligible for this study were summarized and compared between our cohort and the GALLIUM cohort in Table 1. Our cohort included 1,938 eligible patients with FL1-3a from seventeen centers in China, who were randomly assigned to the training and internal validation sets at a 2:1 ratio. The GALLIUM cohort included 1,115 eligible patients with FL1-3a for external validation. With a median follow-up of 31 months (range 29–33 months), the proportion of patients who had POD24 in our cohort was 19.7% (*n* = 383), which was higher than the 14.8% observed in the GALLIUM cohort (*n* = 165; *P* = 0.0006).

**Table 1:**
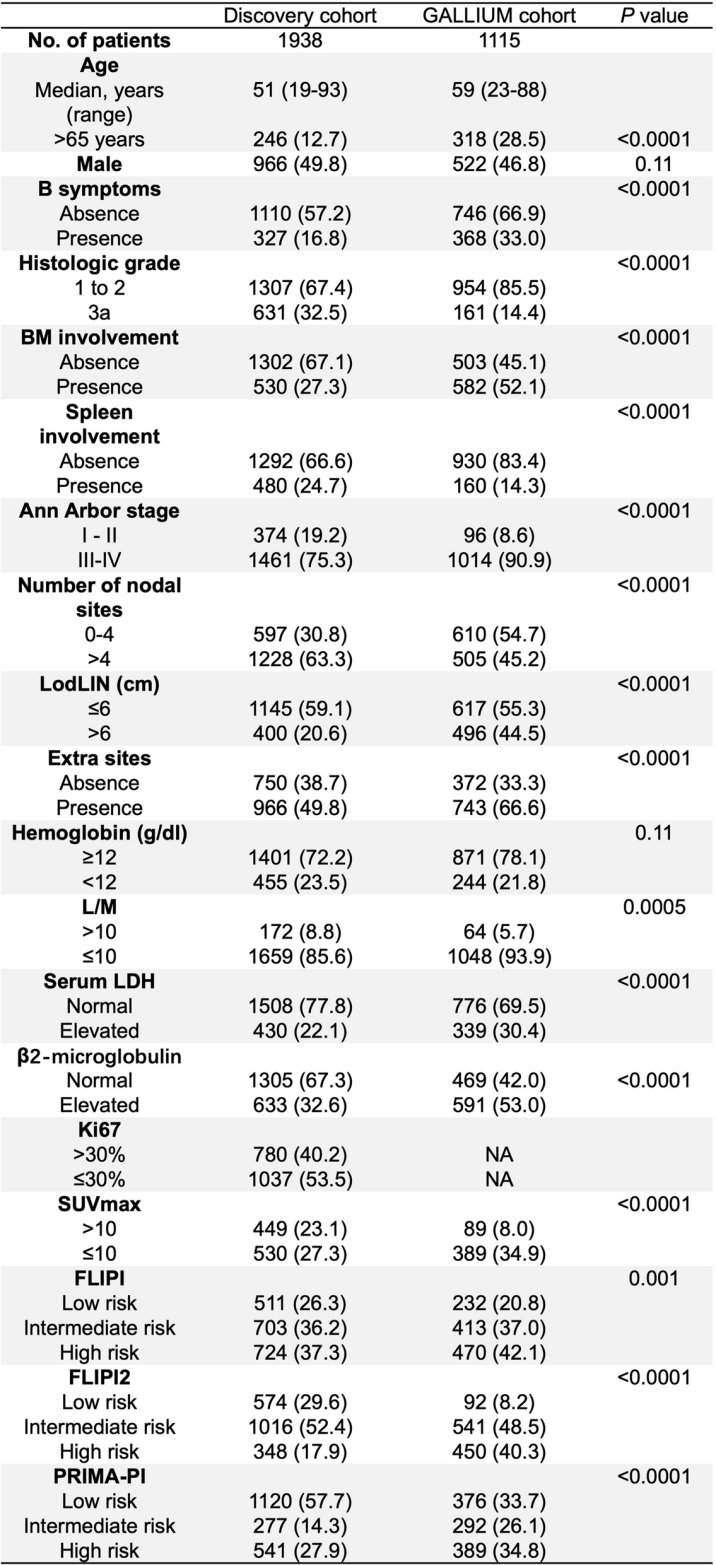
Demographic and clinical characteristics of patients in the discovery cohort and external validation cohorts

The median age of patients in our cohort was 51 years (range, 18 - 93 years), younger than that of the GALLIUM cohort (59 years; range, 23–88 years). In terms of tumor burden, the GALLIUM cohort had a higher proportion of patients with elevated serum levels of LDH and β2-MG, advanced stage diseases (III/IV), and bulky disease (*P* < 0.05 for each). In contrast, the GALLIUM cohort had a lower percentage of patients with splenic involvement compared to our cohort. Together, there are considerable differences between our cohort (for model development and internal validation) and the GALLIUM cohort (for external validation), which might be helpful for generalizing the findings involving the POD24-predicting model (FLIPI-C) proposed in this study.

### Development of the FLIPI-C using a machine learning approach

**Fig. 1:**
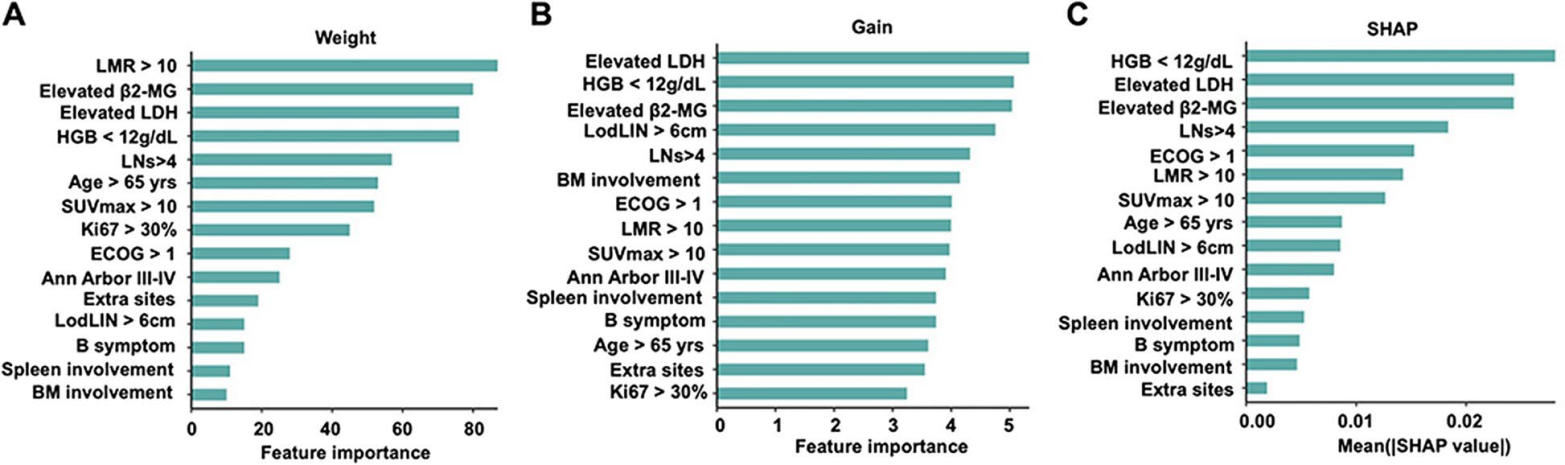
Critical variables with feature importance plots and SHAP values for predicting POD24. **A-C** Summary plot of feature importance including feature weight (**A**), mean gain (**B**) and SHAP value (**C**) of the XGBoost-based prediction model in the training cohort

**Fig. 2:**
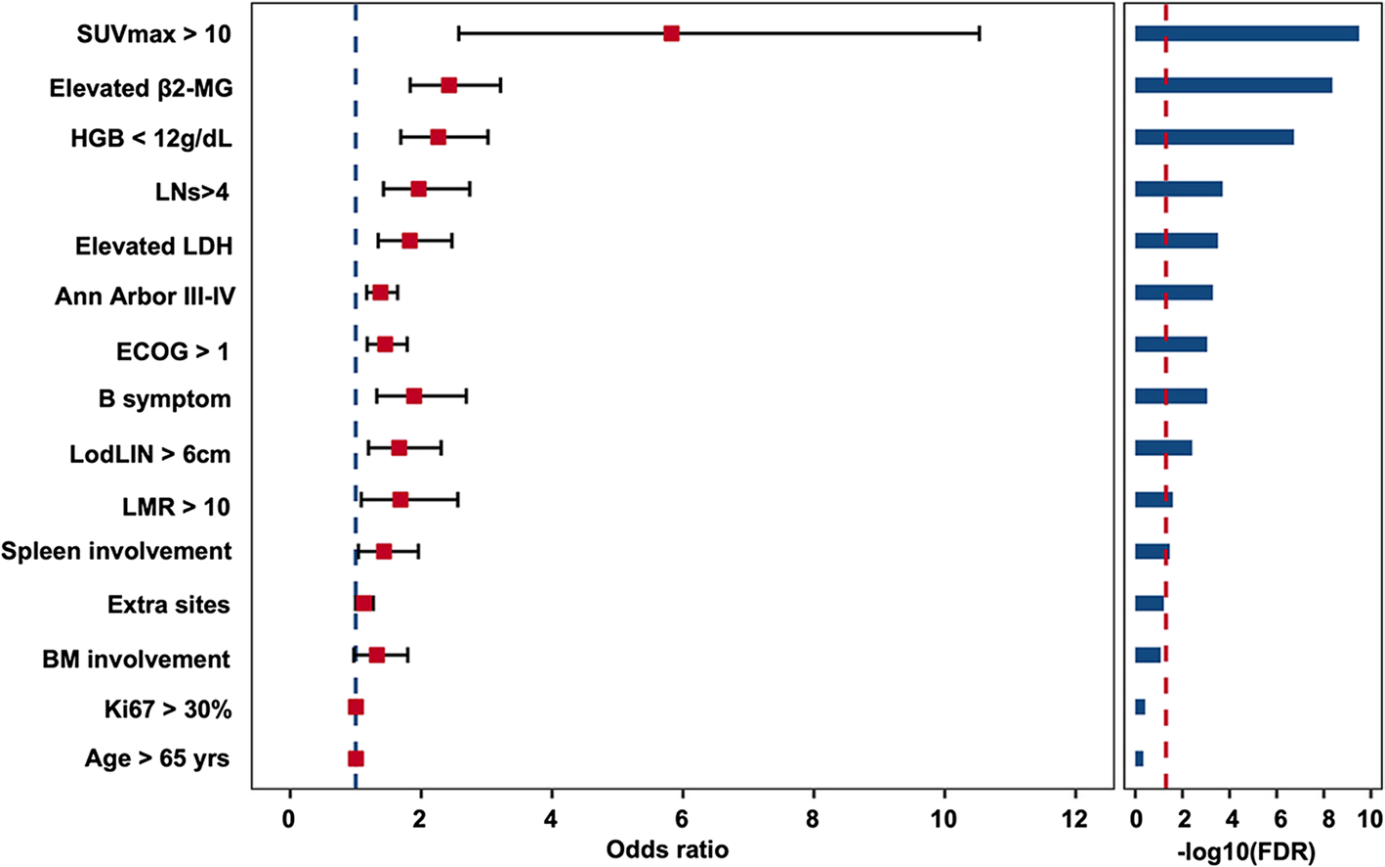
Forest plot of output from the logistic regression model evaluating odds for POD24 risk. The variables identified in the final model, with the corresponding odds ratio and confidence intervals, are shown in the figure

We first compared the ability of different binary classifier algorithms (including DecisionTree, RandomForest, RidgeClassifier, and XGBoost) in predicting POD24 and found that XGBoost achieved the best performance and accuracy (Supplementary Fig. 1), which was then used to develop the FLIPI-C model. Fifteen baseline clinical features at initial diagnosis were inputted into the XGBoost algorithm for screening preliminary variables. The Weight, Gain, and SHAP analyses were performed to obtain the feature importance of each variable for predicting the risk of POD24, and the features were listed in a descending order according to the importance (Fig. 1). To minimize the possibility of overfitting, the XGBoost-derived variables underwent a stepwise-forward selection process. In parallel, logistic regression analysis identified significant predictors of POD24, including SUVmax > 10, elevated β2-MG, Hb < 12 g/dl, involved LNs > 4, LDH > ULN, advanced diseases (stage III/IV), ECOG ≥ 2, B symptoms, and bulky disease (Fig. 2). Also, Cox regression analysis was performed to identify overlapped significant predictors of POD24 (Supplementary Fig. 2).

The XGBoost-based model (FLIPI-C) incorporated six variables that were the most significantly associated with the risk of POD24, including LMR > 10, elevated β2-MG, LDH > ULN, Hb < 12 g/dl, involved LNs > 4, and SUVmax > 10. Based on the weight of the importance, a score was assigned to each of these six variables (LMR, 2 points; other variables, 1 point for each), resulting in a total score of 7 points (Table 2). The performance of this model was evaluated using 5-fold and 10-fold cross-validation, showing an accuracy of 80.6% and 80.8%, respectively.

**Table 2:**
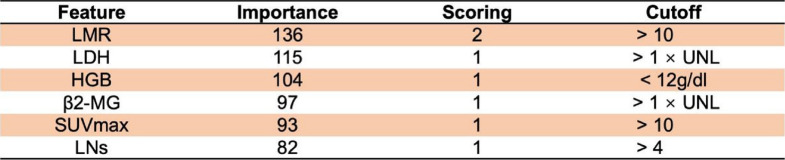
Feature importance for each of six parameters for our trained XGBoost model

### Internal and external validation of the FLIPI-C

The performance of the FLIPI-C was then validated internally in the validation set and externally in the GALLIUM cohort, respectively. In the GALLIUM cohort, 944 patients with complete data required for the FLIPI-C were eventually included for external validation.

**Fig. 3:**
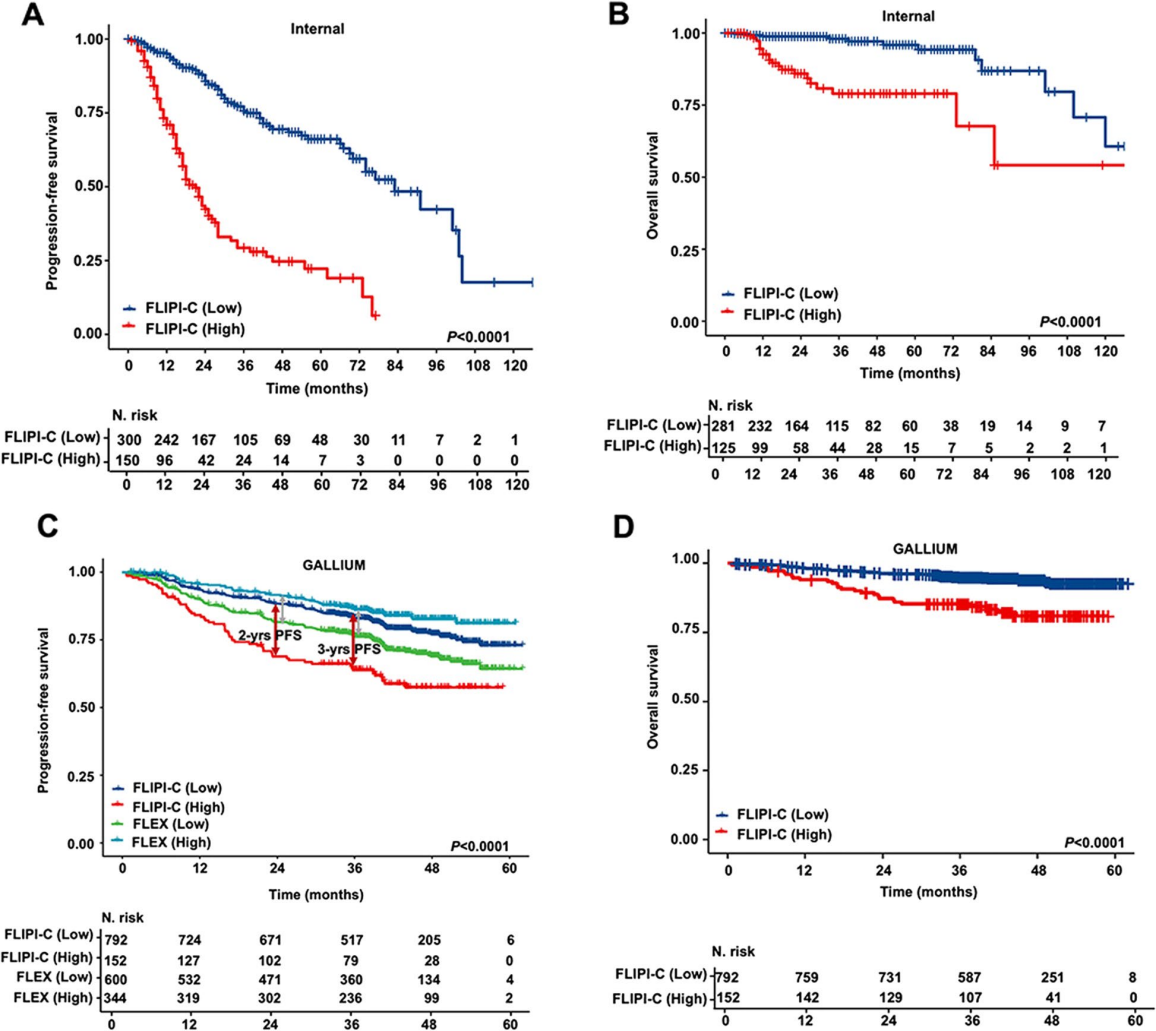
Survival analysis according to FLIPI-C risk score. **A-B** Kaplan–Meier survival analysis of PFS (**A**) and OS (**B**) for patients stratified by FILPI-C risk in the internal validation cohort. **C** Kaplan–Meier survival analysis of PFS for patients stratified by FILPI-C risk compared with by FLEX risk in the external validation cohort. **D** Kaplan–Meier survival analysis of OS for patients stratified by FILPI-C risk in the external validation cohort

The FLIPI-C stratified patients with FL1-3a into low- and high-risk groups, with the optimal cut-off value of 3 points determined by the ROC curve using the Youden Index. This cut-off value displayed the highest sensitivity and specificity for predicting POD24 in the validation set. In the internal validation set, the low-risk group (0–3 points) had a 2-year PFS rate of 85.7% (95% CI, 0.81–0.90) and a 5-year OS rate of 95.8% (95% CI, 0.92–0.99), significantly higher than that of the high-risk group (4–7 points), which had a 2-year PFS rate of 42.4% (95% CI, 0.34–0.52; *P* < 0.0001) and a 5-year OS rate of 78.9% (95% CI, 0.70–0.88; *P* < 0.0001) (Fig. 3A-B**)**. Similar results were obtained in the GALLIUM cohort, with a 2-year PFS rate of 88.4% (95% CI, 0.86–0.90) vs. 63.8% (95% CI, 0.56–0.72; *P* < 0.0001) and a 5-year OS rate of 92.8% (95% CI, 0.90–0.95) vs. 81.0% (95% CI, 0.74–0.88; *P* < 0.0001) for the low- and high-risk groups, respectively. Notably, the differences in the 2-year PFS rate between the low- and high-risk groups defined by the FLIPI-C were more significant than those observed with the FLEX model in both cohorts (Fig. 3C-D).

### Comparison of the predicting accuracy between the FLIPI-C model and existing models

**Fig. 4:**
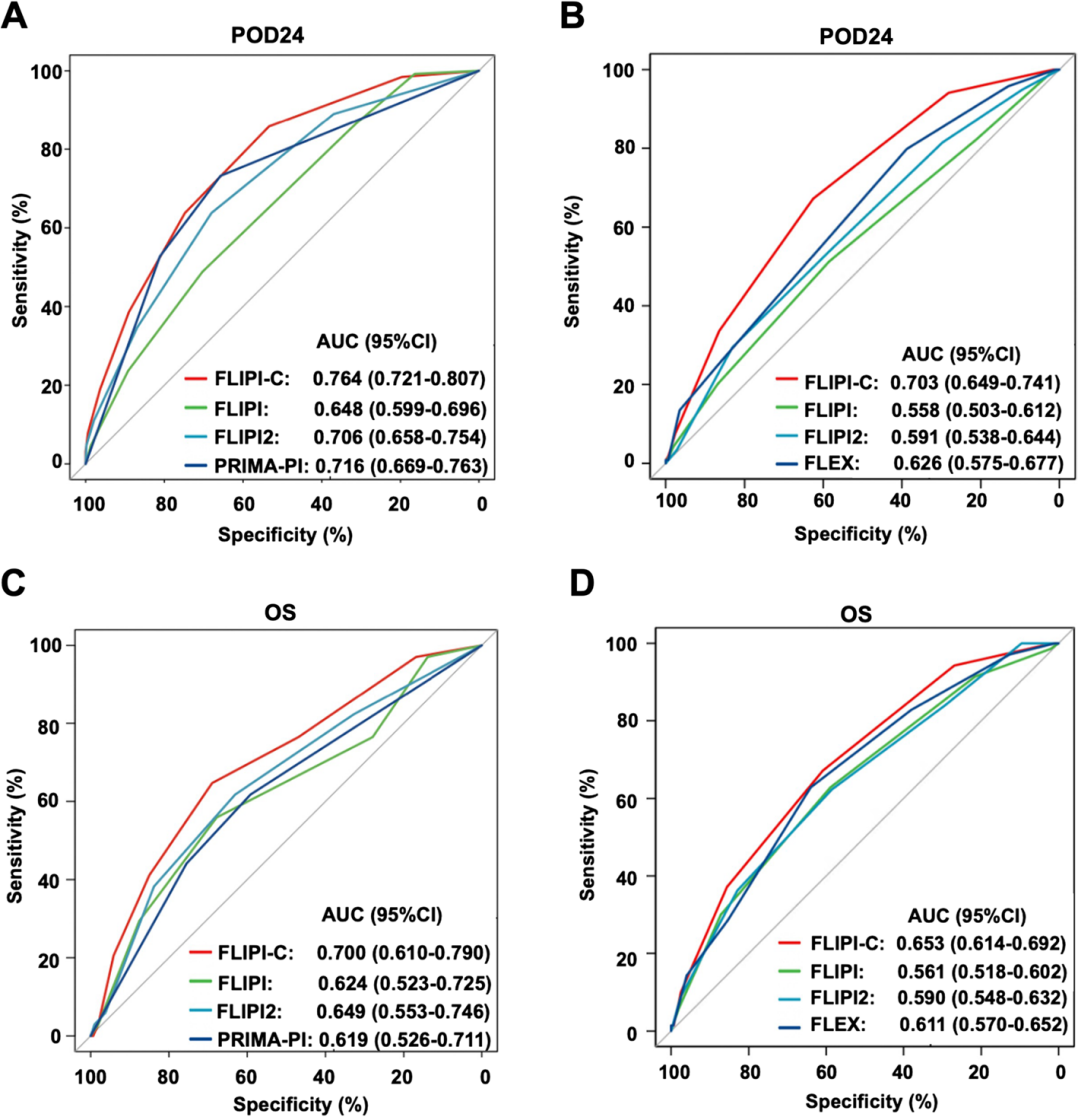
Comparison of the predictive accuracy between the new ML model and previous established models(**A-D**). **A-B** The area under curve (AUC) for POD24 prediction comparing the FLIPI-C model with FLIPI, FLIPI2, and PRIMA-PI models in the internal validation cohort (**A**), and with FLIPI, FLIPI2, and FLEX models in the external validation cohort (**B**); **C-D** The AUC for OS prediction comparing the FLIPI-C model with FLIPI, FLIPI2, and PRIMA-PI models in the internal validation cohort (**C**), and with FLIPI, FLIPI2, and FLEX models in the external validation cohort (**D**)

We then examined whether the accuracy of the FLIPI-C in predicting POD24 and OS would be superior to that of existing prognostic models. In the internal validation cohort, the FLIPI-C had a higher AUC value (0.764; 95% CI, 0.72–0.81) for predicting POD24, compared to the FLIPI (0.648; 95% CI, 0.59–0.69), FLIPI-2 (0.706; 95% CI, 0.65–0.75), and PRIMA-PI (0.716; 95% CI, 0.66–0.76) (Fig. 4A). Consistently, the AUC value of the FLIPI-C for predicting POD24 (0.703; 95% CI, 0.64–0.74) was higher than the FLIPI (0.558; 95% CI, 0.50–0.61), FLIPI-2 (0.591; 95% CI, 0.53–0.64), and FLEX (0.626; 95% CI, 0.57*–*0.67) in the GALLIUM cohort (Fig. 4B). For predicting 3-year OS, the AUC value of the FLIPI-C model was 0.700 (95% CI, 0.61–0.79) and 0.581 (95% CI, 0.53–0.61) in our internal validation set and the GALLIUM cohort respectively, with better performance than the FLIPI, FLIPI-2, PRIMA-PI, and FLEX (Fig. 4C-D).

**Fig. 5:**
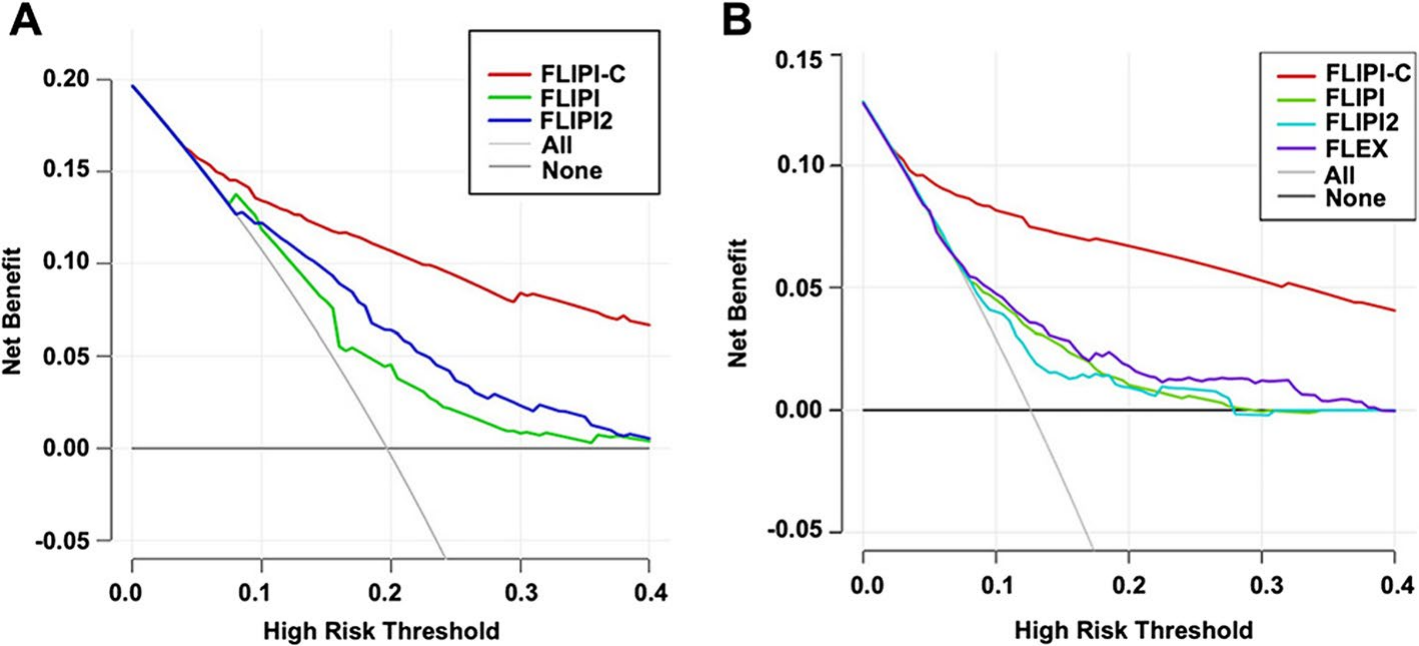
Comparison of decision curve analysis for predicting POD24 between the new ML model and previous established models (**A-B**). **A** Decision curve analysis (DCA) of three prognostic models (FLIPI-C, FLIPI, FLIPI-2) for predicting POD24 in the internal validation cohort. **B** DCA of four prognostic models (FLIPI-C, FLIPI, FLIPI-2 and FLEX) for predicting POD24 in the external validation cohort

Moreover, DCA revealed that the FLIPI-C had the highest net benefit when compared to the existing models (FLIPI, FLIPI-2, PRIMA-PI, and FLEX) in both the internal validation set and the GALLIUM cohort, further supporting its superiority in the stability and reliability of predicting the outcomes in FL patients (Fig. 5A).

### Impact of first-line treatment on risk stratification by the FLIPI-C

**Fig. 6:**
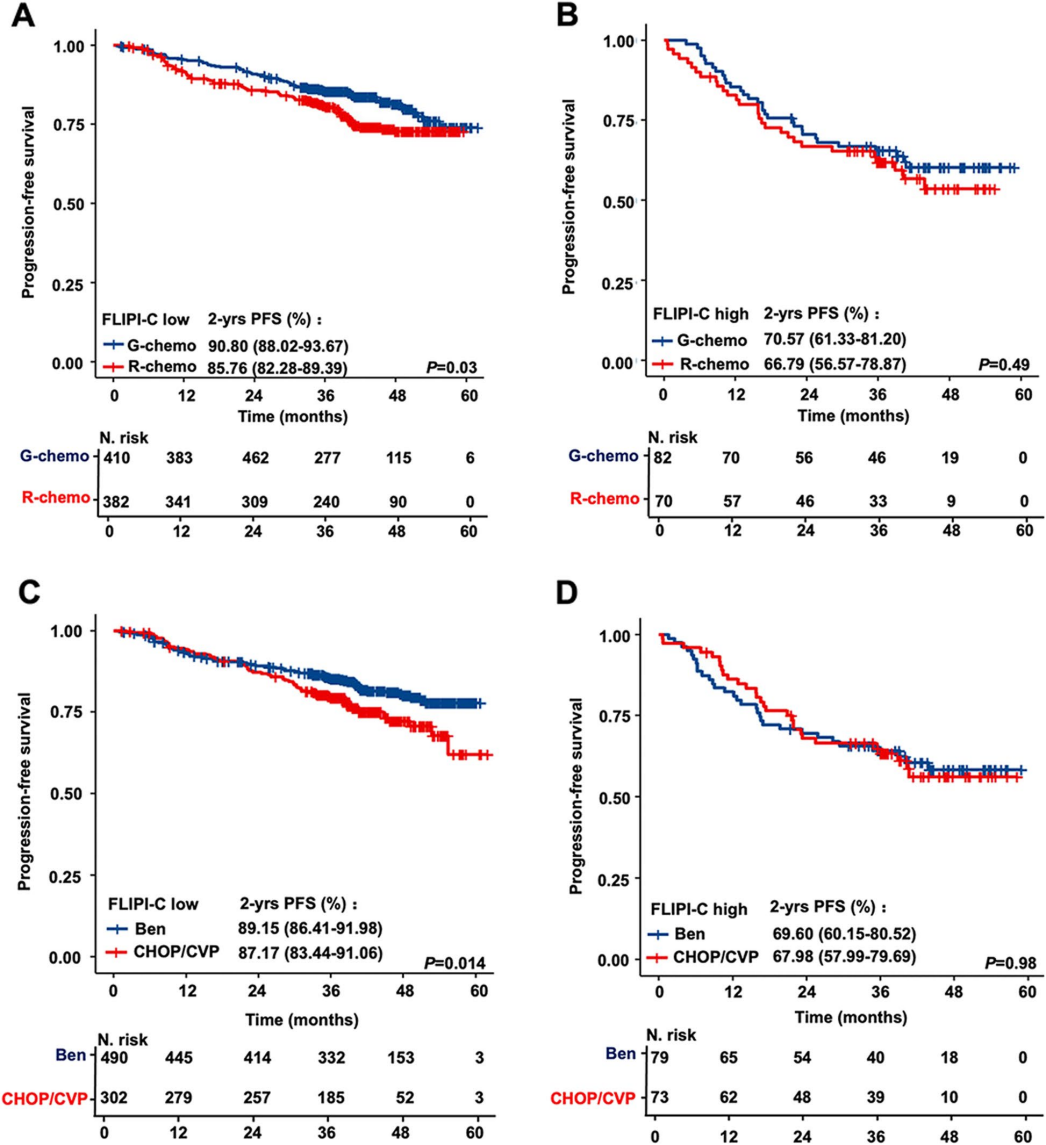
Comparison of progression-free survival of patients depending on the first-line treatment received using the FLIPI-C risk score. **A-B** Kaplan-Meier analysis of PFS for patients who received G-chemo and R-chemo treatment in the FLIPI-C low-risk group (**A**) or in the FLIPI-C high-risk group (**B**). **C-D** Kaplan-Meier analysis of PFS for patients receiving bendamustine or CHOP/CVP plus anti-CD20 therapy (rituximab or obinutuzumab) in the FLIPI-C low-risk group (**C**) or FLIPI-C high-risk group (**D**)

In the GALLIUM cohort (*n* = 944), patients received different first-line therapies, including rituximab-based immunochemotherapy (R-chemo) (*n* = 452; 47.8%), obinutuzumab-based immunochemotherapy (G-chemo) (*n* = 492; 52.1%), bendamustine plus rituximab or obinutuzumab (B-R/G) (*n* = 569; 60.2%), and cyclophosphamide, doxorubicin, vincristine, and prednisone (CHOP) or cyclophosphamide, vincristine, and prednisone (CVP) plus R/G (*n* = 375; 39.7%). Comparing with R-chemo, G-chemo was associated with superior PFS in low-risk patients defined by the FLIPI-C (2-year PFS, 90.8% vs. 85.7%, *P* = 0.03; Fig. 6A). However, no significant differences were observed in high-risk patients (2-year PFS, 70.6% vs. 66.8%, *P* = 0.49; Fig. 6B), in association with a higher rate of POD24 as predicted by the FLIPI-C (Supplementary Fig. 3). Low-risk patients receiving B-R/G had superior PFS to those receiving CHOP or CVP plus R/G (2-year PFS, 89.1% vs. 87.1%, *P* = 0.014; Fig. 6C), while no significant differences were observed in high-risk patients (2-year PFS, 69.6% vs. 67.9%, *P* = 0.98; Fig. 6D). In addition, bendamustine-based immunochemotherapy seemed to have an inferior impact on OS in low-risk patients (5-year OS, 91.3% vs. 95.2%, *P* = 0.035, Supplementary Fig. 4C).

## Discussion

In this study, we developed and validated a novel machine learning-based model combining clinical and imaging parameters to predict POD24 after first-line treatment in patients newly diagnosed with FL1-3a. We evaluated four different machine learning algorithms and found that the XGBoost based-model displayed the best performance in predicting POD24. This model (FLIPI-C) included six independent risk factors most significantly associated with the risk of POD24, identified by the XGBoost algorithm: LMR > 10, LDH > ULN, low Hb, elevated β2-MG, SUVmax > 10, and involved LNs > 4. The FLIPI-C accurately predicted the risk of POD24 in patients with FL1-3a, with improved discriminating ability and the greatest net benefit compared to the existing prognostic models. Moreover, external validation of the model using the independent GALLIUM cohort, which had a distinct demographic and clinical profile, demonstrated robust performance across diverse populations, reinforcing the generalizability of the FLIPI-C model.

Despite differences in demographic characteristics—such as age, disease stage, and baseline biomarker levels—between the Chinese cohort and the GALLIUM cohort, FLIPI-C consistently performed well. The Chinese cohort was younger (median age 51 years) compared to the GALLIUM cohort (median age 59 years), which also had a higher proportion of patients with elevated LDH, β2-MG, advanced-stage disease, and bulky disease, all associated with poorer outcomes. These differences highlight important variations in baseline characteristics that could influence the generalizability of the FLIPI-C model across different populations. While FLIPI-C performed robustly in both cohorts, we acknowledge that demographic factors, such as age, comorbidities, and treatment regimens, may introduce variability in predictive accuracy when applying the model to diverse patient populations. Further validation in more varied, real-world cohorts is necessary to confirm the broad applicability of FLIPI-C in patients with different clinical profiles.

POD24 has been widely recognized as an independent predictor of unfavorable outcomes in patients with FL [5, 22]. While a considerable number of patients with FL experience early relapse or disease progression, it is essential to identify patients with high risk of POD24 and, accordingly, to make timely treatment decision to delay or even prevent relapse or progression [23–26]. The FLIPI, which is widely used in clinical practice, was developed in the pre-rituximab era and categorizes patients with FL into low, intermediate, and high-risk groups with regard to PFS and OS [11]. Although the FLIPI still holds its prognostic value in patients with FL who receive rituximab-based immunochemotherapy [2, 27], it is not optimal for predicting POD24, with false-positive prediction in 44% of all patients. Subsequently, several prognostic models, such as FLIPI-2 and PRIMA-PI, have been developed to identify patients with poor outcomes [13, 28], while their capability for predicting POD24 has not been rigorously evaluated. Recently, genetic alterations have been incorporated into conventional models, leading to an improvement in their performance to estimate the risk of POD24 in more diverse patient populations. For instance, the m7-FLIPI including the mutations of multiple genes (EZH2, ARID1A, MEF2B, EP300, FOXO1, CREBBP, and CARD11), the POD24-PI including specific gene mutations (EP300, FOXO1 and EZH2), and a 23-gene expression signature score have been shown to increase the predicting accuracy [12, 14, 15]. However, the availability of the genetic information largely limits their utilization in daily practice. In contrast, the FLIPI-C offers a pratical solution by using six simple, widely available markers and an easy calculation. DCA demonstrates that FLIPI-C offers a higher net benefit than existing models (FLIPI, FLIPI-2, PRIMA-PI, and FLEX) in both internal and external validation cohorts. The model’s greatest clinical utility lies in the 20–40% risk threshold, where the benefits of treatment outweigh the harms. FLIPI-C effectively identifies high-risk patients who may benefit from more aggressive treatment while avoiding unnecessary interventions for low-risk patients.

A few prognostic models combining clinical and imaging parameters have been reported previously in patients with FL [8, 29]. For example, the FLEX was developed in a cohort of patients with advanced FL in the GALLIUM study [25], which was then validated in the SABRINA study [30]. The FLEX includes nine components, including male, sum of the products of lesion diameters (SPD) in the highest quartile, grade 3a, extranodal site > 2, ECOG PS > 1, low Hb (< 12 g/dl), elevated β2-MG, low NK cell count (< 100/ul) in peripheral blood, and elevated LDH. It particularly emphasizes the predictive role of SPD and NK cell count, which are however relatively difficult to be obtained in daily practice. While the internal validation demonstrated better performance of the FLEX in predicting POD24 than other models (e.g., FLIPI, FLIPI-2 and PRIMA-PI), the external validation did not verify these results [8]. Thus, further investigation is needed to validate and generalize the POD-predicting capability of the FLEX. It is also worth noting that the FLEX was developed using Cox proportional hazards regression that has potential limitations in the analysis of non-linear outcomes or data with missing variables, which may thus affect its predicting accuracy in certain scenarios.

Considering the limitations of the existing prognostic models, a more reliable and practicable model in predicting POD24 is needed for patients with FL. Taken advantage of machine learning methods that have been shown accurate in predicting adverse events and flexible in handling non-linear outcomes and missing data [16, 31], we utilized a machine learning approach (XGBoost) to develop a POD24-predicting model for patients with FL1-3a, aiming to improve the accuracy of predicting the outcome of this heterogeneous population. In addition to the clinical characteristics included in the existing models, the XGBoost algorithm also identified LMR and SUVmax, an imaging feature from PET examination, as independent predictors of POD24, consistent with previous studies [32–36]. While PET examination may not be available for all patients with newly diagnosed FL due to cost considerations, the FLIPI-C performed well in both our cohort and the GALLIUM cohort, even without PET data. This also suggests that the FLIPI-C might retain its capability to predict POD24 and OS of patients with FL1-3a cross different ethnic populations.

The FLIPI-C developed in this study for predicting the risk of POD24 could identify high-risk patients with FL who need more intensive and effective treatment. This is particularly important for patients who relapsed and experienced POD24 after first-line treatment, as alternative therapies such as lenalidomide/rituximab, phosphoinositide 3-kinase inhibitors, autologous stem cell transplantation, and CAR-T are likely to benefit this subset of patients [37, 38]. On the other hand, early identification of high-risk patients at the time of diagnosis could help optimizing initial treatment (i.e., risk-adapted therapy) to reduce or even prevent POD24 [39, 40]. In this context, several trials (e.g., GALLIUM and NCI NCTN) have evaluated different therapeutic strategies. The GALLIUM study (phase III) has demonstrated that obinutuzumab chemotherapy (G-chemo) provides a superior long-term benefit (PFS) to R-chemotherapy for previously untreated FL patients [41], and also significantly reduces the cumulative incidence of POD24 [42]. In the GALLIUM cohort, we observed that G-chemo significantly improved PFS in low- but not high-risk patients defined by the FLIPI-C. This differential response to G-chemo between low- and high-risk patients may reflect differences in disease biology. Low-risk patients typically have less aggressive disease, with a more favorable microenvironment and lower tumor burden, which allows for a better response to standard therapies like G-chemo. In contrast, high-risk patients often have more aggressive disease, with higher tumor burden, elevated biomarkers (e.g., LDH, β2-MG), and poorer prognostic factors, potentially leading to intrinsic resistance to G-chemo. Additionally, genetic mutations or immune checkpoint alterations may further reduce the effectiveness of standard treatments in this group. These factors likely explain why G-chemo benefits low-risk patients more than high-risk ones. However, caution should be taken when interpreting this observation, as more high-risk patients would experience POD24. Nonetheless, these findings highlight the importance of choosing an appropriate initial therapy to reduce the risk of early progression and improve outcomes in patients with FL.

In summary, we propose a novel prognostic model for predicting POD24 in FL patients, demonstrating considerable accuracy and consistency across entirely different ethnic populations. This model was developed using a machine learning approach, which consists of six baseline characteristics most significantly associated with the risk of POD24. In addition to the factors (elevated β2-MG, LDH > ULN, Hb < 12 g/dl, and involved LNs > 4) included in the existing prognostic models (e.g., FLIPI, FLIPI-2), LMR > 10 and SUVmax > 10 were also identified as independent predictors of POD24. The FLIPI-C outperformed existing models, such as FLIPI, FLIPI2, PRIMA-PI, and FLEX, in predicting both POD24 and OS. With improved accuracy in risk stratification, the FLIPI-C may guide treatment decision-making more precisely for FL patients with heterogeneous outcomes, especially those at high risk of POD24 who may benefit from more intensive therapies. Early identification of FL patients with high risk of POD24 (e.g., using the FLIPI-C) at diagnosis is essential to prevent POD24, an ultimate goal for the treatment of FL, through individualizing risk-adapted therapy for first-line treatment. Therefore, the FLIPI-C proposed in this retrospective study warrants further validation in prospective settings.

## Supplementary Information

Supplementary Material 1: Figure 1. Comparison of accuracy in predicting POD24 between different algorithms, including DecisionTree, RandomForest, RidgeClassifier and XGBoost.

Supplementary Material 2: Figure 2. Univariate analyses of cancer-specific survival in the training cohort. (A-B) Univariate analyses of progression-free survival (PFS, A) and overall survival (OS, B) in the training cohort.

Supplementary Material 3: Figure 3. Comparison of the rate of POD24 identified by FLIPI-C with FLIPI, FLIPI-2 and FLEX in the external validation cohort.

Supplementary Material 4: Figure 4. Comparison of overall survival of patients after receiving first-line treatment according to FLIPI-C risk score. (A-B) Kaplan-Meier survival analysis of OS for patients treated with G-chemo and R-chemo treatment in the FLIPI-C low-risk group (A) or in the FLIPI-C high-risk group (B). (C-D) Kaplan-Meier survival analysis of OS for patients receiving bendamustine or CHOP/CVP plus anti-CD20 therapy (rituximab or obinutuzumab) in the FLIPI-C low-risk group (C) or in the FLIPI-C high-risk group (D).

## Data Availability

No datasets were generated or analysed during the current study.
