# Supplementary figures and images for: A machine learning-based model to predict POD24 in follicular lymphoma: a study by the Chinese workshop on follicular lymphoma
Source: Biomark Res. 2025 Jan 3;13:2. doi: 10.1186/s40364-024-00716-4 (PMC11697473; doi:10.1186/s40364-024-00716-4)

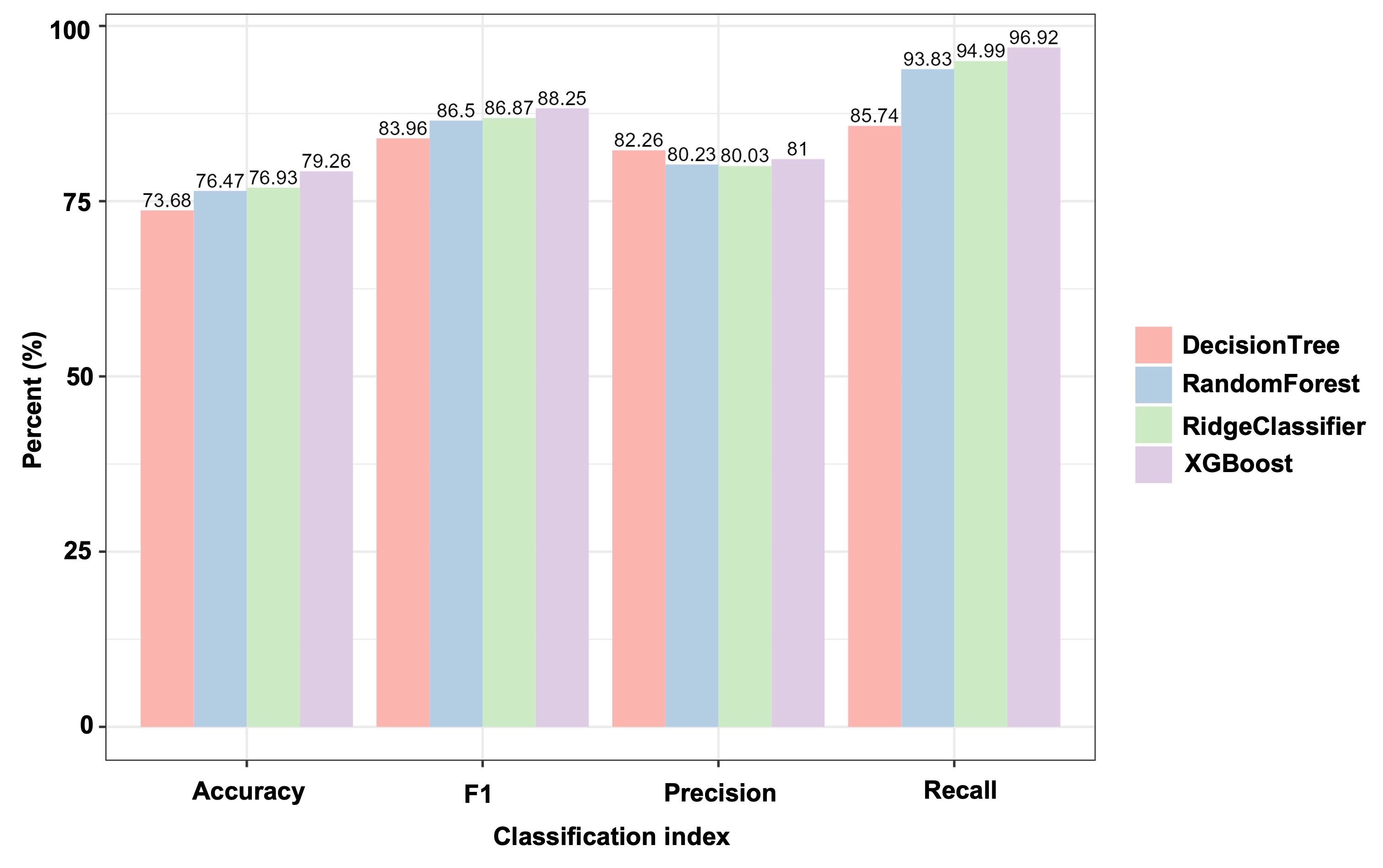

Supplement: Supplementary file 1 — Supplementary Material 1: Figure 1. Comparison of accuracy in predicting POD24 between different algorithms, including DecisionTree, RandomForest, RidgeClassifier and XGBoost. [file 40364_2024_716_MOESM1_ESM.jpg]

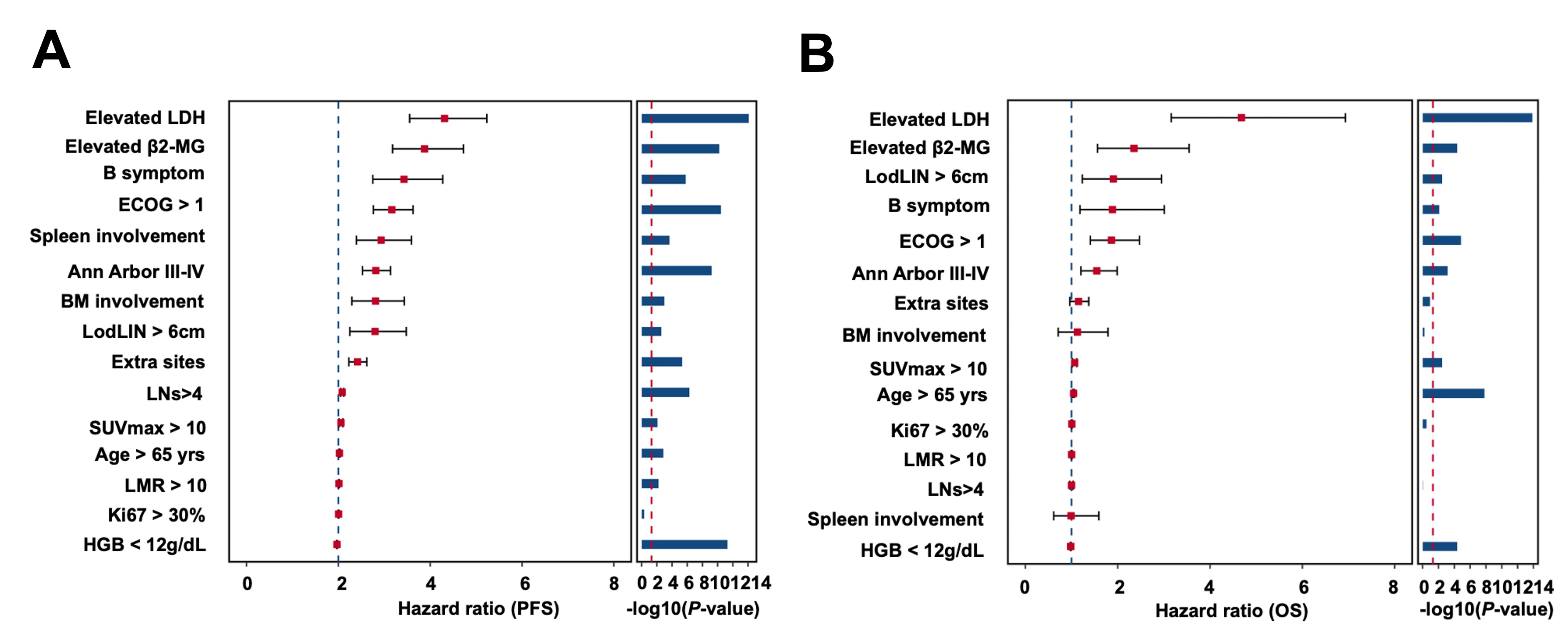

Supplement: Supplementary file 2 — Supplementary Material 2: Figure 2. Univariate analyses of cancer-specific survival in the training cohort. (A-B) Univariate analyses of progression-free survival (PFS, A) and overall survival (OS, B) in the training cohort. [file 40364_2024_716_MOESM2_ESM.jpg]

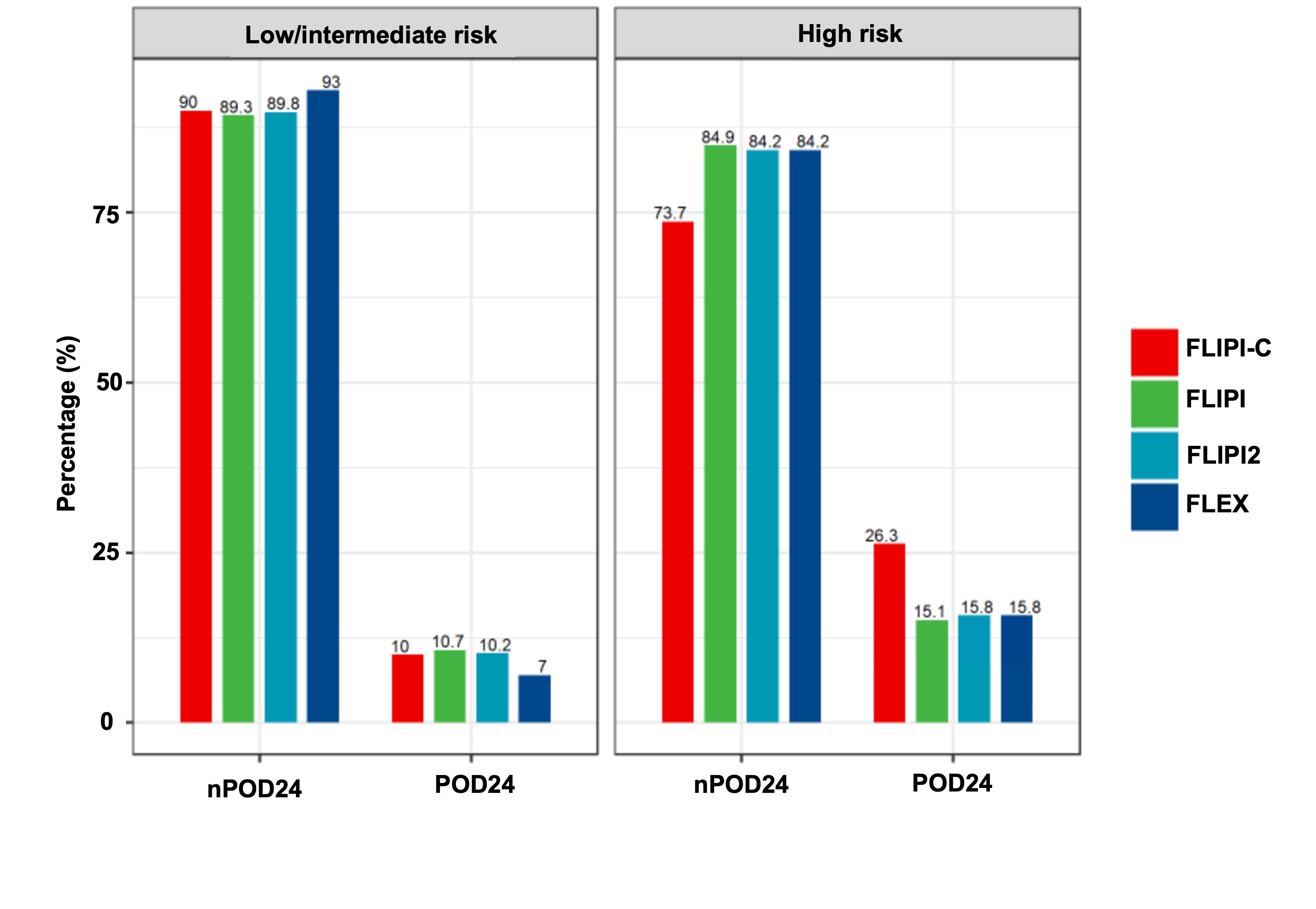

Supplement: Supplementary file 3 — Supplementary Material 3: Figure 3. Comparison of the rate of POD24 identified by FLIPI-C with FLIPI, FLIPI-2 and FLEX in the external validation cohort. [file 40364_2024_716_MOESM3_ESM.jpg]

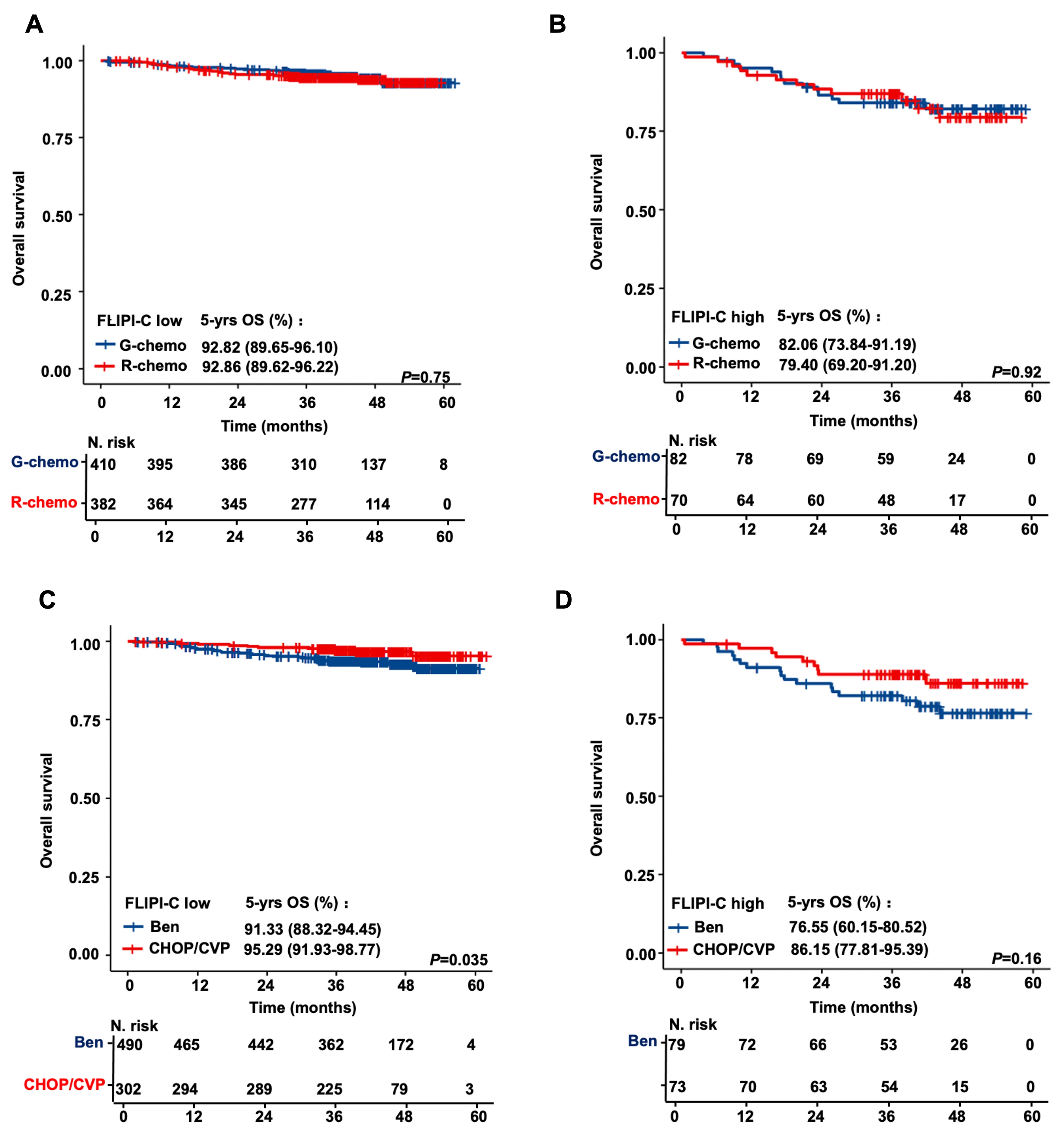

Supplement: Supplementary file 4 — Supplementary Material 4: Figure 4. Comparison of overall survival of patients after receiving first-line treatment according to FLIPI-C risk score. (A-B) Kaplan-Meier survival analysis of OS for patients treated with G-chemo and R-chemo treatment in the FLIPI-C low-risk group (A) or in the FLIPI-C high-risk group (B). (C-D) Kaplan-Meier survival analysis of OS for patients receiving bendamustine or CHOP/CVP plus anti-CD20 therapy (rituximab or obinutuzumab) in the FLIPI-C low-risk group (C) or in the FLIPI-C high-risk group (D). [file 40364_2024_716_MOESM4_ESM.jpg]
